# Hair analysis for detection of triptans occasionally used or overused by migraine patients—a pilot study

**DOI:** 10.1007/s00228-016-2074-5

**Published:** 2016-05-31

**Authors:** Anna Ferrari, Carlo Baraldi, Manuela Licata, Daniele Vandelli, Filippo Marchesi, Federica Palazzoli, Patrizia Verri, Cecilia Rustichelli, Enrico Giuliani, Enrico Silingardi

**Affiliations:** 1 Unit of Medical Toxicology, Headache Centre and Drug Abuse, Department of Diagnostic, Clinical and Public Health Medicine, University of Modena and Reggio Emilia, Policlinico, Via del Pozzo, 71, 41124 Modena, Italy; 2 Forensic Toxicology Laboratory, Department of Diagnostic Medicine, Department of Diagnostic, Clinical and Public Health Medicine, University of Modena and Reggio Emilia, Via del Pozzo, 71, 41124 Modena, Italy; 3 Department of Life Sciences, University of Modena and Reggio Emilia, via G. Campi, 103, 41125 Modena, Italy; 4 Anaesthesiology, Department of Diagnostic, Clinical and Public Health Medicine, University of Modena and Reggio Emilia, Via del Pozzo, 71, 41124 Modena, Italy; 5 Unit of Forensic Medicine, Department of Diagnostic, Clinical and Public Health Medicine, University of Modena and Reggio Emilia, Via del Pozzo, 71, 41124 Modena, Italy

**Keywords:** Hair analysis, Monitoring, Migraine, Medication-overuse headache, ROC curve, Triptan

## Abstract

**Purpose:**

The aim of this study is to evaluate the detection rate of almotriptan, eletriptan, frovatriptan, sumatriptan, rizatriptan, and zolmitriptan in the hair of migraineurs taking these drugs; the degree of agreement between type of self-reported triptan and triptan found in hair; if the concentrations in hair were related to the reported cumulative doses of triptans; and whether hair analysis was able to distinguish occasional use from the overuse of these drugs.

**Methods:**

Out of 300 headache patients consecutively enrolled, we included 147 migraine patients who reported to have taken at least one dose of one triptan in the previous 3 months; 51 % of the patients overused triptans. A detailed pharmacological history and a sample of hair were collected for each patient. Hair samples were analyzed by liquid chromatography-electrospray tandem mass spectrometry (LC-MS/MS) by a method that we developed.

**Results:**

All the triptans could be detected in the hair of the patients. The agreement between type of self-reported triptan and type of triptan found in hair was from fair to good for frovatriptan and zolmitriptan and excellent for almotriptan, eletriptan, sumatriptan, and rizatriptan (*P* < 0.01, Cohen’s kappa). The correlation between the reported quantities of triptan and hair concentrations was statistically significant for almotriptan, eletriptan, rizatriptan, and sumatriptan (*P* < 0.01, Spearman’s rank correlation coefficient). The accuracy of hair analysis in distinguishing occasionally users from overusers was high for almotriptan (ROC AUC = 0.9092), eletriptan (ROC AUC = 0.8721), rizatriptan (ROC AUC = 0.9724), and sumatriptan (ROC AUC = 0.9583).

**Conclusions:**

Hair analysis can be a valuable system to discriminate occasional use from triptan overuse.

**Electronic supplementary material:**

The online version of this article (doi:10.1007/s00228-016-2074-5) contains supplementary material, which is available to authorized users.

## Introduction

Migraine is a chronic disorder with recurrent attacks that affects 10 % of the adult population [1]. The effective treatment of attacks is fundamental to quickly relieve suffering and prevent the progression toward chronic migraine [2]. Triptans, selective agonists at 5-hydroxytryptamine 1B/1D (5-HT_1B/1D_) receptor subtype, are recommended as first-line drugs for the acute treatment of the attack in patients suffering from moderate-severe migraine [3]. However, like every medication, triptans must be taken correctly to be effective. Their use in clinical practice has instead revealed various problems [4]. On the one hand, underuse both by episodic [5] and chronic migraine [6] patients has often been reported [7]. On the other hand, there is a large number of patients overusing these medications, developing a type of medication-overuse headache (MOH) [8, 9], defined as “triptan-overuse headache” [10]. This chronic headache is precisely due to “regular intake of one or more triptans in any formulations, on ten or more days per month for >3 months” [10]. MOH is a serious, disabling, and difficult to treat disorder; only the withdrawal of the overused medication can lead to an improvement [9]. To face this situation, the ideal thing would be to be able to monitor triptan use [8]. To monitor drug use, indirect or direct methods can be employed [11]. Indirect methods, such as self-report and diaries, overestimate adherence [12]. Direct methods, such as dosage of triptans in blood and urine, are unfit, reflecting only recent drug intake.

In recent years, hair has become a fundamental biological specimen to monitor adherence to pharmacological treatments [13, 14] and document objectively the progress of detoxification programs [15–17]. Hair analysis has a very wide window of time of detection, which depends on the length of the hair; therefore, you only need few samples to monitor a long period. In addition, hair analysis allows the clinician to distinguish between occasional and chronic use [15, 17].

The aim of our study was to apply hair analysis to clinical practice by a method that we validated [18], in order to evaluate the detection rate of triptans on the market in Italy in the hair of migraine patients taking these drugs, the degree of agreement between type of self-reported triptan and type of triptan found in hair, whether the concentrations measured in hair reflected the cumulative doses of triptans that the patients had reported to have taken, and whether hair analysis was able to distinguish occasional use from the overuse of these drugs.

## Patients and methods

### Patients

Of a total of 300 headache patients consecutively coming to the Headache Centre of the University Hospital of Modena (Italy), 147 patients (mean age ± SD: 45.29 ± 11.3 years, *F* 96 %) who had reported to have used in the previous 3 months at least one dose of one triptan by any way of administration and whose hair in the nuchal area was at least 5 cm long, took part in the study (all demographic data are available as supplementary table). According to ICHD-3beta criteria [10], they were divided into two groups: (1) with occasional triptan use and (2) with triptan overuse (regular intake of one or more triptans in any formulations, on ten or more days per month for >3 months). According to ICHD-3beta criteria [10], all triptan overusers had been diagnosed with chronic migraine. Among the patients taking triptans occasionally, 50 (70 %) had been diagnosed with migraine without aura, 12 (7 %) with chronic migraine, 5 (7 %) with migraine with and without aura, and 4 (6 %) with migraine with aura. Seventy-eight percent of the patients was between 25 and 55 years old.

All the patients had given their written consent to their participation in the study. They were enrolled from October 1st, 2013 to December 23rd, 2014.

### Procedures

For each patient, we collected by a specific form the anagraphic data, diagnosis of headache, hair characteristics (color and cosmetic treatments), and pharmacological history. According to international guidelines for hair analysis [19], a hair sample of at least 7 mm in diameter and 4 cm in length was taken from each patient’s nuchal area. From each hair sample, we cut and analyzed a single section measuring 3 cm, proximal, i.e., near the scalp, to cover the previous 3 months.

The concentrations of almotriptan, eletriptan, frovatriptan, rizatriptan, sumatriptan, and zolmitriptan in hair samples were determined by liquid chromatography-electrospray tandem mass spectrometry (LC-MS/MS). The method had been developed by us and validated [18] according to the model proposed by the Scientific World Group for Forensic Toxicology in 2013, in Standard Practices for Method Validation in Forensic Toxicology [19]. A number was assigned to each hair sample. The laboratory made blind assessments.

### Data analysis

A descriptive analysis and a comparison between triptan occasional users and overusers were conducted as far as the following aspects were concerned: demographic characteristics, headache diagnosis, and pharmacological history.

The results of the detection of triptans in hair were then compared to the patients’ self-report regarding the occasional use or overuse of these drugs. The concentrations measured in hair were considered *positive expected* if in agreement with the self-reported occasional use or overuse of triptans; *positive unexpected* if they were not in agreement with the self-reported occasional use or overuse of triptans; *negative expected* if the patient had taken at most four defined daily doses (DDDs) of each triptan (almotriptan 50 mg, eletriptan 160 mg, frovatriptan 10 mg, sumatriptan 200 mg, rizatriptan 40 mg, and zolmitriptan 10 mg) in the previous 3 months; and *negative unexpected* if the patient had taken more than four DDDs of each triptan in the previous 3 months.

The agreement between the self-reported occasional use or overuse of triptans and the concentrations measured in hair had been assessed on the basis of previous data [18], considering that overuse was shown by levels >105 pg/mg for almotriptan, >500 pg/mg for eletriptan, >4.5 pg/mg for frovatriptan, >60 pg/mg for rizatriptan, >55 pg/mg for sumatriptan, and >18 pg/mg for zolmitriptan. Unexpected results and triptans found in hair, but not self-reported, were considered as signs of non-adherence by patients and excluded from further statistical analysis.

Excluding results of non-adherent patients, we analyzed the relationship between the cumulative doses and hair concentrations of each triptan and compared the mean cumulative doses reported and the hair concentrations of each triptan between the two groups of patients. Finally, we determined the accuracy of hair analysis in detecting triptan overusers and cut-off hair concentrations to discriminate triptan overuser from occasional user.

### Statistical analysis

Statistical analysis was conducted by the StataIC 13 software. The continuous variables normally distributed were expressed as mean ± standard deviation and dichotomous variables were indicated as percentage. The comparison between the means was investigated by Student’s *t* test for independent data; to compare binary variables, Chi-square test or one-sided Fisher’s exact test was used when appropriate.

The relationship between two continuous variables was analyzed by Spearman’s *R* correlation coefficient. The agreement between the type of triptan reported by the patient and the one detected by hair analysis was analyzed by Cohen’s *K* coefficient. *P* < 0.05 was chosen as significant for all the tests.

The predictive accuracy of hair analysis was quantified by the area under the curve (AUC) on receiver-operating characteristic (ROC) curve analysis, the greater the area under the curve the better hair analysis performs. The best cut-point discriminating overusers from occasionally users were determined using the Liu’s method. Sensitivity, specificity, negative predictive value (NPV), and positive predictive value (PPV) of hair analysis were determined at every cut-point through ROC curve analysis**.**

## Results

The vast majority (Table 1) of the analysis conducted on the hair samples of the 147 patients who had declared to have taken one or more triptans in the previous 3 months detected measurable concentrations of each triptan reported by the patients, both by occasional users and overusers.

**Table 1:** Positive results (triptan detected) in hair analysis for each triptan self-reported by 147 patients (since various patients used more than 1 triptan, the triptans reported were 176 in total)

Triptan	Number (%) of positive occasional users in hair test/number of self-reported triptan users	Number (%) of positive overusers in hair test/number of self-reported triptan users	Total number (%) of positive results in hair test/total number of self-reported triptan users
Almotriptan	23/25 (92)	15/15 (100)	38/40 (95)
Eletriptan	12/15 (80)	29/31 (93.5)	41/46 (89)
Frovatriptan	1/10 (10)	3/5 (60)	4/15 (26.6)
Rizatriptan	20/20 (100)	8/8 (100)	28/28 (100)
Sumatriptan	11/18 (61)	20/22 (91)	31/40 (77.5)
Zolmitriptan	2/5 (40)	2/2 (100)	4/7 (57.1)
Total	69/93 (74)	77/83 (93)	146/176 (83)

The agreement between the type of triptan self-reported and type of triptan detected in hair, analyzed by Cohen’s kappa, was statistically significant (*P* < 0.01) for each triptan, excellent (kappa over 0.75) for almotriptan (kappa 0.88), eletriptan (kappa 0.87), rizatriptan (kappa 0.94), and sumatriptan (kappa 0.76), and fair to good (kappa from 0.40 to 0.75) for frovatriptan (kappa 0.49) and zolmitriptan (kappa 0.72).

More than 70 % of almotriptan, eletriptan, and rizatriptan hair concentrations (Table 2) were *positive expected*, i.e., in agreement with the self-reported occasional use or overuse of the triptan in the previous 3 months. *Positive unexpected* concentrations in comparison with the self-reported occasional use or overuse of triptan were the minority. Unreported triptans were only found in very few samples, in particular rizatriptan and sumatriptan, each of which was found in 5 out of 147 patients (3.5 %), almotriptan, found in 4 patients (3 %), and eletriptan, found in 3 (2 %) (scatterplots of all analytical data are available as supplementary figure).

**Table 2:** Results of the comparison between hair concentration of each triptan and the self-reported occasional use or overuse of that triptan in the previous 3 months

Results of hair analysis for every determined triptan							
Results	Almotriptan *n* = 40 (%)	Eletriptan *n* = 46 (%)	Frovatriptan *n* = 15 (%)	Rizatriptan *n* = 28 (%)	Sumatriptan *n* = 40 (%)	Zolmitriptan *n* = 7 (%)	Total *n* = 176 (%)
*Positive expected*	30 (75)	35 (76)	3 (20)	20 (71)	23 (58)	2 (28.5)	113 (64)
*Positive unexpected*	8 (20)	6 (13)	1 (7)	8 (29)	8 (20)	2 (28.5)	33 (19)
*Negative expected*	2 (5)	3 (7)	2 (13)	0 (0)	5 (12)	0 (0)	12 (7)
*Negative unexpected*	0 (0)	2 (4)	9 (60)	0 (0)	4 (10)	3 (43)	18 (10)

The agreement between the cumulative doses of each triptan that the patients had reported to have taken in the previous 3 months, and their respective hair concentrations with *expected* results was statistically significant for each triptan (almotriptan: 0.79, eletriptan: 0.70, rizatriptan: 0.8, and sumatriptan: 0.58; *P* < 0.01, Spearman’s rank correlation coefficient), except frovatriptan and zolmitriptan, because of the very few cases.

The analysis of each group’s results (Table 3) indicated that in the previous 3 months, overusers had taken as an average significantly higher cumulative doses of each triptan than occasional users (*P* < 0.01, Student’s *t* test). Mean hair concentrations of each triptan in overusers were also significantly higher, at least four times (*P* < 0.05, Student’s *t* test) compared to occasional users, excepted for frovatriptan and zolmitriptan. The 95 % CIs of average hair concentrations were very wide.

**Table 3:** Mean cumulative doses (mg) that triptan occasional users and overusers had reported to have taken in the previous 3 months and respective mean concentrations (pg/mg) in hair (the 113 *positive expected* results were analyzed)

Triptan	Occasional users (*n* = 71) Mean (95 % CI)		Overusers (*n* = 76) Mean (95 % CI)	
	3-month cumulative doses (mg)	Hair level (pg/mg)	3-month cumulative doses (mg)	Hair level (pg/mg)
Almotriptan	113.75 ** 66.19÷161.31	165.55 * 106.50÷224.60	1055 574.60÷1535.40	1306.5 176.14÷2436.90
Eletriptan	453.33 ** 274.05÷632.62	371 ** 161.23÷558.77	3447.69 2245.74÷4649.65	2470.73 1760.39÷3181.07
Frovatriptan	29.16 ** 13.79÷44.54	0.56 −0.73÷1.84	137.91 105.81÷170.02	4.25 −1.16÷9.66
Rizatriptan	43.08 * 24.55÷61.60	20.62 * 9.28÷31.95	925.71 47.96÷1803.47	541.29 75.36÷1007.21
Sumatriptan	826.73 ** 454.04÷1199.42	83.18 ** 17.34÷149.03	7919.17 3421.15÷12,417.18	352.92 160.29÷545.54
Zolmitriptan	26.5 ** −3.32÷56.32	18.6 −21.73÷58.93	1087.5 −2247.88÷4422.88	26 −37.53÷ 89.53

Occasional users vs. overusers: 3-month cumulative doses and hair level, *P < 0.05; **P < 0.01, Student’s *t* test for unpaired data

**Fig. 1:**
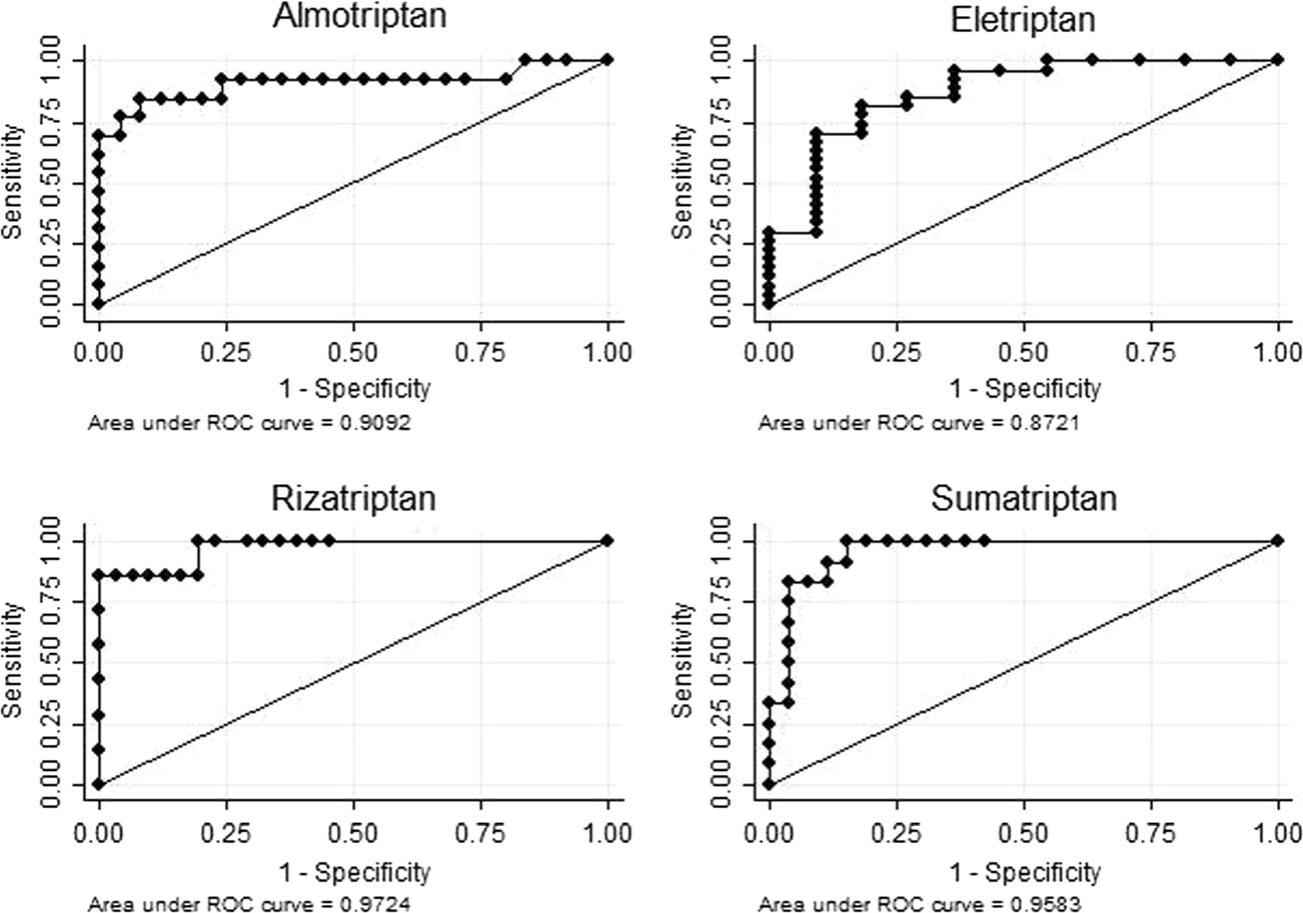
Receiver operator characteristic (ROC) curve for almotriptan, eletriptan, rizatriptan, and sumatriptan

The largest area under the ROC curve (Fig. 1), indicating excellent accuracy in correctly identifying triptan overusers, was observed for rizatriptan; however, every ROC curve analysis showed a good accuracy of hair analysis in detecting triptan overusers also for almotriptan, eletriptan, and sumatriptan. In particular, at the cut-point for rizatriptan (Table 4), the specificity was 100 % with a positive predictive value of 100 %.

**Table 4:** Cut-points of hair concentration to distinguish occasional from triptan overusers calculated by Liu method applied to ROC curve analysis

Triptan	Cut-point (ng/mg)	Sensitivity (%)	Specificity (%)	Positive predictive value (PPV) (%)	Negative predictive value (NPV) (%)
Almotriptan	526	77	96	91	89
Eletriptan	789	82	82	91	75
Rizatriptan	179	86	100	100	97
Sumatriptan	139	83	96	90	89

## Discussion

The results of our study (Table 1) showed that six triptans could be determined in the hair of the patients who had occasionally used or overused them to treat their migraine attacks during the previous 3 months. As a whole, reported triptans were detected in 83 % of hair analyses. There was a substantial concordance between the types of triptans reported by the patients and those objectively detected by hair analysis. The agreement between the type of self-reported triptan and the one detected in hair was statistically significant (*P* < 0.01) and more than fair to excellent for each of these drugs, according to Cohen’s *k* values. In spite of this, the concordance between self-report and hair analysis was incomplete as far as the cumulative doses taken were concerned. Indeed, 29 % of hair analyses (Table 2) gave *unexpected* results (both positive, 19 %, and negative, 10 %), i.e., inadequate with the self-reported occasional use or overuse of the triptan. Furthermore, unreported triptans were detected in few samples. These results, considered as signs of non-adherence, are consistent with those of other studies, which found out approximately or more than 35 % of non-adherence to pharmacological treatments in migraine patients [2, 7, 20].

Triptans have an indolic structure, are mainly basic, and have a large distribution volume and a certain lipophilicity [21]. All these characteristics allow their penetration into the systemic circulation of growing hair, where the pH is acid (approximately 4) [17], and their accumulation in the keratin matrix. Hair analysis, traditionally used in the forensic field [22], is more and more used in clinical medicine and research for drug monitoring with undoubted advantages [13, 14, 17], among which the possibility to determine the concentrations of more drugs simultaneously. This opportunity is particularly useful for migraine patients, who often take various types of medications to treat acute attacks. With the method that we developed [18], it was possible to determine the concentrations of each of the six triptans simultaneously in the same hair sample.

In normal clinical practice, the physician formulates the diagnosis of MOH and proposes the treatment according to what the patient reports or records in his/her diary, without having an objective datum about drug overuse. By the analysis of a single hair sample of 4 cm, we were able to prove objectively the intake of each triptan during the previous 3 months, which is precisely the reference period in order to diagnose medication-overuse headache, according to the ICHD-3 beta classification [10]. Measuring in blood or urine has instead a short surveillance window: it shows at most the previous 2–3 days. With these matrixes, we would have needed a very large number of samples for each individual patient to monitor 3 months, causing an unacceptable trouble to the patients.

The ICHD-3 beta classification [10] defines overuse in terms of frequency, and regularity of use during at least 3 months. By hair analysis, it was possible to translate the cumulative doses taken by the patients into measurable concentrations in the keratin matrix. Both the cumulative doses of triptans taken (Table 3) and hair average concentrations were significantly higher (*P* < 0.05, Student’s *t* test) in overusers than in occasional users. It does not therefore seem to exist a process of saturation of the absorption of triptans in the keratin matrix.

Approximately, half the patients of our sample used triptans to treat their migraine attacks occasionally. By hair analysis, we also detected the concentrations of each triptan which were associated with this appropriate use. With traditional matrixes, this would have been impossible, because these medications are present for a short time in blood and urine after intake [23]. They have a plasma half-life which goes from 2–3 h for sumatriptan, rizatriptan, and zolmitriptan, to 4–5 h for eletriptan; only frovatriptan has a plasma half-life of 25 h [21].

The kinetics of orally administered triptans has a significant interindividual variability, depending on various factors such as reduced and erratic absorption during the migraine attack and variations according to sex and metabolism [24]. When determining hair concentrations of triptans taken orally, more variability factors add, such as cosmetic hair treatment, hair color, or melanin [17], which can have influenced the trapping of triptans in the keratin matrix. As a matter of fact, the confidence intervals of average triptan concentrations in hair (Table 3) were very wide, both for occasional users and overusers. All triptans have the same mechanism of action. The main differences concern pharmacokinetics and metabolism [24]. Precisely, these differences, together with the different lipophilicity and pKa value, are reflected in large differences of concentrations found in hair between a triptan and another. In fact, drugs penetration in hair depends on their pKa values and polarity. The lower lipophilicity, the lower incorporation into hair and high pKa values increase the drug melanin binding [17]. In fact, even with roughly similar cumulative doses, hair concentrations of eletriptan, the triptan considered the most lipophilic of all, are much higher than those of the less lipophilic sumatriptan and with low oral bioavailability, of around 14 % [21].

Considering only adherent-patients, there was a statistically significant relationship (*P* < 0.01) between the cumulative doses taken and the concentrations in hair for each triptan (when the number of cases was adequate). A relationship between doses and hair concentrations has also been described for carbamazepine [25], antiretrovirals [14, 26], and imatinib [27] and it represents a key characteristic for the use of hair analysis in therapeutic drug monitoring.

Our study has some limits. It was a sample with a wide female prevalence, partly because of female prevalence in migraine and even more in chronic migraine associated to drug overuse [9], from which suffered approximately 50 % of the patients enrolled. In spite of this, we do not have any elements to suppose significant differences between female and male subjects in the penetration of triptans into hair. The sample was deliberately heterogeneous for the type of triptans used, the frequency of use, and the presence of outpatients and inpatients, in order to assess the applicability of hair analysis in the most different clinical conditions. All the patients only took the medications orally. However, oral intake is the favorite modality and the most used by the majority of migraine patients [9]. As far as we know, the present study is the first to compare self-reported triptan taking and triptan concentrations in hair, so we cannot compare it to others. As a precaution, we have therefore considered as *expected* the negative results of the few patients who had taken less than four DDDs of each triptan in the previous 3 months. The choice of four DDDS for 3-month period was arbitrary in the absence of other standard references and more sensitivity analyses will serve to support it.

Adherence to triptan prescriptions is fundamental to get their therapeutic benefit [2]. Patients using triptans should therefore be checked every 3–6 months [8]. Our results suggest that hair analysis can be a valuable system to monitor the use of triptans. In fact, hair analysis accuracy in detecting triptan overuse (Fig. 1) was excellent for almotriptan, rizatriptan, and sumatriptan, and was good for eletriptan. The cut-point concentrations (Table 4) better discriminating occasional users from overusers were associated with high positive predictive values, indicating that a false positive result is rare.

In conclusion, hair analysis is a unique approach to monitor prescription triptan adherence according to the recommended procedure to check migraine patients every 3–6 months [3, 8]. Moreover, the determination of triptan concentrations in hair could be useful to make the response to prophylaxis objective in clinical trials.

We hope that our findings can pave the way for future research and application of hair analysis for the monitoring of triptan use/adherence in migraine management.

### Authors’ contribution

All authors participated in study design, performed research, data analysis, and drafting of manuscript content. All authors reviewed and approved the manuscript.

## Electronic supplementary material

ESM 1 (DOC 103 kb)
