# Supplementary material for: Hair analysis for detection of triptans occasionally used or overused by migraine patients—a pilot study
Source: Eur J Clin Pharmacol. 2016 May 31;72:1075–81. doi: 10.1007/s00228-016-2074-5 (PMC4980420; doi:10.1007/s00228-016-2074-5)
Supplement: Supplementary file 1 — (DOC 103 kb) [file 228_2016_2074_MOESM1_ESM.doc]

**Demographic data**

| Variable | Occasional users  n (%) | Overusers  n (%) | Total  n (%) |
| --- | --- | --- | --- |
| Number of patients | 71 (49) | 76 (51) | 147 (100) |
| Mean age ± SD (years) | 43.13 ± 10.05 | 47.32 ± 12.23* | 45.29 ± 11.31 |
| Range (years) | 18-74 | 18-73 | 18-74 |
| Females | 67 (94) | 74 (95) | 141 (96) |
| *Ethnia* |  |  |  |
| Caucasic | 70 (99) | 75 (99) | 145 (99) |
| African | 1 (1) | 1 (1) | 2 (1) |
| *Education* |  |  |  |
| University degree | 11 (15) | 23 (30)** | 34 (23) |
| Primary, secondary, high school | 60 (46) | 53 (43) | 113 (77) |
| *Occupation* |  |  |  |
| Employed | 49 (69) | 61 (80) | 110 (75) |
| Unemployed | 22 (31) | 15 (20) | 37 (25) |
| *Marital status* |  |  |  |
| Married | 40 (56) | 54 (71) | 94 (64) |
| Unmarried | 31 (44) | 22 (29) | 53 (36) |
| *Setting* |  |  |  |
| Outpatients | 64 (90) | 38 (50)** | 102 (69) |
| Inpatients | 7 (10) | 38 (50) | 45 (31) |
| *Drugs used for attack treatment* | |  | |
| Only triptans | 13 (18) | 14 (18) | 27 (18) |
| A single type of triptan | 65 (92) | 56 (74)** | 121 (82) |
| Two types of triptan | 6 (8) | 16 (21)** | 22 (15) |
| Three or more types of triptan | 0 (0) | 4 (5) | 4 (3) |

Occasional users *vs* overusers: **P*<0.05, Student’s *t* test for unpaired data; ***P*<0.05, Fisher’s exact test

**Scatter-plots of all analytical data ( cumulative doses taken in the previous 3 months and hair concentration for every triptan).**

**
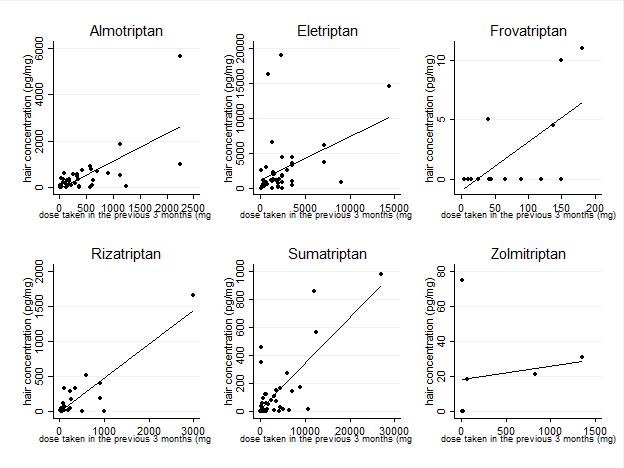
**
